# Neurofilament light chain as a biomarker for acute hepatic porphyrias

**DOI:** 10.3389/fneur.2024.1384678

**Published:** 2024-04-18

**Authors:** Paulo Sgobbi, Paulo de Lima Serrano, Bruno de Mattos Lombardi Badia, Igor Braga Farias, Hélvia Bertoldo de Oliveira, Alana Strucker Barbosa, Camila Alves Pereira, Vanessa de Freitas Moreira, Ícaro França Navarro Pinto, Acary Souza Bulle Oliveira, Wladimir Bocca Vieira de Rezende Pinto

**Affiliations:** Division of Neuromuscular Diseases, Department of Neurology and Neurosurgery, Federal University of São Paulo (UNIFESP), São Paulo, Brazil

**Keywords:** porphyria, neurofilament, biomarker, acute hepatic porphyria, inherited metabolic disorders, inborn errors of metabolism

## Abstract

**Background:**

Acute hepatic porphyrias (AHP) represent a rare group of inherited metabolic disorders of heme biosynthesis pathway. This study aims to determine the diagnostic and prognostic value of serum neurofilament light chain (NfL) as potential biomarker for AHP.

**Methods:**

We conducted a cross-sectional observational study to evaluate NfL levels in patients with AHP. They were divided in different groups: normal health individuals; patients with definitive diagnosis of AHP during acute episodes; patients with AHP and infrequent attacks; patients with AHP and recurrent attacks; asymptomatic individuals with positive genetic testing and urinary delta-aminolevulinic acid (ALA) and porphobilinogen (PBG) levels elevated 4 or more times (“high excretors”); asymptomatic individuals with exclusive positive genetic test; control group with Hereditary Amyloidosis related to Transthyretin with Polyneuropathy (ATTRv-PN).

**Results:**

During acute attacks, serum NfL levels were 68 times higher compared to normal controls and disclosed a strong correlation with ALA and PBG levels; also exhibited elevated levels in patients with chronic symptoms regardless of the number of disease attacks compared to healthy controls, and at similar levels to patients with ATTRv-PN, which is a model of progressive neuropathy.

**Conclusion:**

This study represents the first to establish NfL as a biomarker for AHP, disclosing NfL as a sensitive biomarker for axonal damage and chronic symptom occurrence. This study not only underscores that neurological damage associated with the disease in any patient, irrespective of the number of attacks, but also reinforces the progressive and profoundly debilitating nature of acute and chronic symptoms observed in individuals with AHP.

## 1 Introduction

Hereditary porphyrias are a rare group of inherited metabolic disorders due to dysfunction in the heme biosynthesis pathway. They can be classified depending on the principal site of accumulation of toxic intermediates, either as *erythropoietic* or *hepatic*. Additionally, they can be categorized based on clinical manifestations as acute porphyrias, characterized by neurovisceral attacks, or chronic porphyrias, which involve prominent cutaneous manifestations in photo exposed skin areas (1, 2).

Acute hepatic porphyrias (AHPs) represents a rare group of four inherited metabolic disorders: acute intermittent porphyria (AIP), variegate porphyria (VP), hereditary coproporphyria (HCP), with autosomal dominant trait inheritance caused by variants in the genes *HBMS*, *PPOX* and *CPOX*. These genes are responsible for encoding the porphobilinogen deaminase, protoporphyrinogen oxidase and coproporphyrinogen oxidase enzymes, respectively. Additionally, ALA (delta-aminolevulinic acid) dehydratase deficiency porphyria (also known as ALAD deficiency or Doss porphyria) is an autosomal recessive disease caused by biallelic variants on *ALAD* gene, which is responsible for the production of delta-aminolevulinate dehydratase enzyme (1, 2).

AHPs are clinically characterized by life-threatening neurological manifestations that can occur either acutely or chronically, resulting in debilitating and progressive neurological impairment. The burden of these manifestations negatively impacts the quality of life (QoL) of patients. In addition, AHPs are associated with severe long-term complications such as hepatocellular carcinoma, chronic kidney disease and hypertension (1, 2).

In AHPs, there is a pronounced overproduction of porphyrins in the liver, leading to the abnormal accumulation of toxic intermediate metabolites, primarily porphobilinogen (PBG) and ALA. This accumulation results in significant dysfunction of the nervous system through multiple mechanisms. These include the dysfunction of neurotransmitter receptors, particularly gamma-aminobutyric acid (GABA), increased production of reactive oxygen species, dysfunction of nitric oxide synthase leading to secondary vasomotor dysfunction causing cerebral and enteral vasospasm, reduction of neuronal mitochondrial oxidative phosphorylation due to the direct compromise of the mitochondrial membranes and the respiratory chain complexes, disruption of axonal membrane with dysfunction of Na^+^–K^+^ ATPase pump, and elevated production of several pro-inflammatory cytokines (e.g., IL-1b, IL-2, IL-6, IL-17, TNF, INF-γ) and vascular endothelial growth factor (VEGF) (3–5).

Patients with AHPs can be categorized into four subgroups: (1) latent genetic mutation carriers who are asymptomatic and biochemically inactive [with normal levels of the porphyrin precursors 5′-aminolevulinic acid (ALA) and porphobilinogen (PBG)], (2) asymptomatic high excretors who do not currently experience acute attacks but exhibit biochemical activity (with increased urinary ALA and PBG ≥4 times the upper limit of normal), (3) sporadic attack patients with infrequent acute episodes (<4 per year), and (4) recurrent attack patients (≥4 per year) (6).

A significant proportion of patients with AHPs, around 65–70% in large cohorts, experience chronic symptoms that markedly impair their quality of life, requiring extensive pharmacological treatment and clinical management, regardless of the number of attacks experienced throughout the course of the disease (7–9).

Patients experiencing recurrent or sporadic attacks in AHPs often report progressive physical and neurological deterioration, including symptoms such as pain, fatigue, depression, anxiety, insomnia, gastrointestinal issues (such as constipation, nausea, vomiting), and neuropathy. Individuals with recurrent attacks also may encounter challenges in adapting to the limitations imposed by disease, negatively impacting their relationships and work activities (7–9).

Neurofilaments are neuron-specific cytoskeletal fibers that constitute the most abundant cytoskeletal component of mature neurons when they organize into fibrillary networks, serving as the principal cytoskeletal component and imparting structural stability while resisting mechanical stress. Categorized as intermediate filaments (IF) based on their diameter (~10 nm), which falls between that of actin filaments (6 nm) and myosin filaments (15 nm), neurofilaments play a crucial role in maintaining the integrity of neurons (10, 11).

There is a growing body of evidence suggesting that NfL may have the capability to detect subclinical neuronal damage even in non-primary neurological diseases, a phenomenon likely associated with the development of short- or long-term neurological consequences. This association between NfL and the degree of neurological impairment and outcomes has been well-established in various medical contexts, including patients admitted to intensive care units (ICU), during the perioperative phase, in cases of sepsis, primary psychiatric disorders (such as depression, bipolar disorder, schizophrenia and anorexia nervosa), and long-term complications of COVID-19. Additionally, a few reports have examined NfL as a biomarker for neuronal injury in conditions resulting from physical agents (such as decompression sickness) and in gynecological and dermatological disorders (12–14).

The pathophysiology of these chronic symptoms in AHPs remains poorly understood, and there exists an unmet medical need for the identification of biomarkers that can assess disease progression and therapeutic response. In this study, our aim is to determine the diagnostic and prognostic value of serum neurofilament light chain (NfL) as a biomarker for AHPs, in addition to clinical evaluation and other assessment tools.

## 2 Materials and methods

### 2.1 Patients and individuals

A cross-sectional observational clinical study was conducted to collect and analyze levels of NfL in patients diagnosed with AHPs. The participants were divided into seven groups as follows: (A) normal health individuals with a negative genetic test for AHPs and no history of neurological disorders, comprising a group of 20 volunteers; (B) patients with a definitive diagnosis of AHP during an acute episode, defined by abdominal pain requiring hospital admission or hemin therapy, or severe neurological manifestations such as acute flaccid paralysis, acute encephalopathy or status epilepticus requiring hospital admission and treatment in the intensive care unit; (C) patients with a definitive diagnosis of AHP classified as “sporadic patients” with infrequent attacks, having less than four attacks in the year prior to study recruitment; (D) patients with a definitive diagnosis of AHP that are classified as “recurrent attack patients,” having experienced more than four attacks in the last year; (E) asymptomatic individuals with a positive genetic test for AHP, exhibiting increased urinary ALA and PBG levels ≥4 times the upper limit of normal, referred to as “high excretors”; (F) asymptomatic individuals with a positive genetic test for AHPs but without evidence of biochemical activity (normal levels of ALA and PBG), known as “latent genetic carriers”; (G) an external control group consisting of patients with hereditary amyloidosis related to transthyretin with polyneuropathy (ATTRv-PN), a condition characterized by prominent peripheral nervous system involvement similar to observed in AHPs (15).

All individuals in control group A underwent assessment by two different neurologists (PS and PLS) with special expertise in neurodegenerative diseases. This evaluation aimed to confirm the absence of any history or clinical signs of neurodegenerative diseases or any other neurological disorders that could justify abnormal values of neurofilament levels.

All participants in groups B, C and D had the diagnosis of AHP confirmed at some point during the course of their disease. This diagnosis was established by an increase in urinary PBG levels exceeding 4 times the upper limit of normal, along with genetic testing that identified a variant in any of the four genes associated with AHPs.

Participants in groups E and F are individuals who, at some point, underwent biochemical analysis for the excretion profile of ALA and PBG in urine and genetic testing for AHPs. This was prompted by a positive family history, involving a family member with a definitive diagnosis of AHP.

All patients in group G underwent evaluation by neurologists (PS and PLS), who confirmed the definitive diagnosis of Hereditary Amyloidosis related to Transthyretin with Polyneuropathy based on symptoms and clinical signs associated with the disease. This confirmation was supplemented by a genetic test identifying a pathogenic variant in the *TTR* gene. At the time of this study, participants in group G were in stage 1 of familial amyloid polyneuropathy (FAP)-Coutinho and had been undergoing regular treatment with tafamidis at a dosage of 20 mg per day for a period longer than 1 year.

Patients undergoing treatment with givosiran or on prophylactic use of heme-derived therapies (hemin) were excluded from this study, as the use of these disease-modifying therapies could interfere with the analysis of NfL levels or introduce biases in the interpretation of results.

All patients gave written informed consent for participation in the study and for this publication, and study procedures were approved by institutional ethics committees (CAAE: 50517321.2.0000.5505).

### 2.2 Clinical assessment

All patients underwent a comprehensive medical evaluation in a single interview, encompassing the review of medical data, clinical and neurological examinations, quality of life assessment, and the referral for blood and urinary sample collection.

Patients in group B were evaluated during acute episodes, involving the clinical characterization of the attack, blood collection for NfL analysis, and urine sample collection to assess ALA and PBG levels.

The clinical assessment of patients in groups C and D included the collection of data such as current age, sex, age at the onset of symptoms, time taken to establish a definitive diagnosis, subtype of AHP, number of attacks during the course of the disease, number of attacks in the last year, psychiatric evaluation for depression and anxiety and assessment of quality of life.

Patients in groups E and F were interviewed to determine their interest in participating in the study, review the results of biochemical and genetic test results performed in the past, and confirm the absence of signs and symptoms related to symptomatic AHP.

The assessment of patients in group G involved collecting of data such as current age, sex, disease duration, FAP Coutinho staging, adherence to tafamidis treatment, and a quality of life assessment.

### 2.3 ALA and PBG analysis

The analysis of ALA was conducted on a spot urinary sample using a spectrophotometer in the visible ultraviolet region (Spectrophotometric UV–Vis). The concentration was assessed by measuring creatinine, and the result was expressed as mg/g of creatinine, with a normal range of <4.5 mg/g creatinine. Porphobilinogen was quantified in a 24 h urine sample using ion-exchange chromatography, with the results expressed as mg/24 h with the normal value below 2.0 mg/24 h.

### 2.4 Genetic test

DNA was extracted from the probands in a clinical setting, utilizing peripheral blood leukocytes or saliva. Exome capture was also carried out in a clinical environment using the Agilent Clinical Research Exome v1, following the manufacturer’s instructions. Sequencing procedures were executed on an Illumina NextSeq platform. The obtained exome data were aligned to the GRCh37.75/hg19 reference genome using the Burrows-Wheeler Aligner (BWA; version 0.7.17-r1188). Identification of variants, including single-nucleotide variants (SNVs) and indels, was conducted in accordance with the best practices of the Broad Institute, employing the Genome Analysis Toolkit (GATK, version 3.8-0-ge9d806836) software, and subsequently annotated using Variant Effect Predictor (VEP, version 88.14). All exomes fulfilled the criterion of a minimum of 95% of target bases covered at a depth greater than 10×.

### 2.5 NfL measurement

Blood samples were collected by venipuncture in ethylenediaminetetraacetic acid (EDTA) tubes for plasma. After centrifugation, plasma samples were aliquoted and stored at −80°C. The concentration of serum NfL was measured using single molecule array (SIMOA) technology and the NF-light assay on an HD-X analyzer, following the instructions provided by manufacturer. All samples were measured in a single round of experiments, utilizing one batch of reagents. The analyses were performed by board-certified laboratory technicians who were blinded to the clinical data.

### 2.6 Psychiatric evaluation

Psychiatric evaluation for depression and anxiety was conducted during the medical interview through the application of two scales: the 8-item Patient Health Questionnaire depression scale (PHQ-8; scale, 0–24) and the 7-item Generalized Anxiety Disorder scale (GAD-7; scale, 0–21). Moderate to severe depression was identified with a PHQ-8 cutoff score of ≥10 (16), and mild, moderate, and severe anxiety was identified with GAD-7 scores of 5, 10, and 15, respectively (17).

### 2.7 Quality of life assessment

The assessment of quality of life involved patients providing descriptions of their health-related quality of life (HRQoL) using the SF-12 Health Survey Version 2 (SF-12v2). This health profile instrument comprises 12 items that assess eight sub-domains classified as physical functioning (PF), role physical (RP), bodily pain (BP), general health (GH), vitality (VT), social functioning (SF), role-emotional (RE) and mental health (MH) (18, 19). These eight sub-domain scores can be weighted and summarized into two component scores: the physical component summary (PCS) score and the mental component summary (MCS) score. The reference value for PCS and MCS scores for the US population have a mean of 50 and a standard deviation of 10, with a lower score indicating a poor health status (18, 19).

### 2.8 Statistical analysis

Descriptive statistics was applied, and categorical variables were summarized using counts and percentages of the total population. Continuous variables were reported using mean and standard deviation (SD). Student’s *t*-test and the chi-squared test or the Fisher exact test were used for correlation of quantitative and qualitative variables, respectively. Correlations between continuous variables were performed with Pearson correlation coefficient, with a correlation coefficient of *r* < 0.3 considered weak, *r* = 0.3–0.59 moderate, and *r* ≥ 0.6 a strong correlation. The software Stata^®^ 17.0 was used for statistical analysis, and a two-sided *p* < 0.05 was considered statistically significant.

## 3 Results

The study encompassed 137 participants, distributed across various groups: 20 individuals in group A, designated as the “healthy control”; 20 in group B; 20 in group C, labeled “sporadic attacks”; 20 in group D, termed “recurrent attacks”; 18 in group E, denoted as “high excretors”; 19 in group F, identified as “latent AHP”; and finally, 20 in group G. For the purposes of this article, the collective term “symptomatic AHP” refers to groups B, C, and D, while groups E and F are categorized as asymptomatic AHP.

Of the 137 patients, 105 (76.6%) are Caucasian, 91 (66.4%) are female, and the mean age during the study period was 35.5 (±10.47). Additionally, the mean age within each group did not exhibit a statistically significant difference. Among the 60 patients diagnosed with symptomatic AHPs, comprising groups B, C and D, 48 (80%) were female and the mean age for each group were 31.35 (±9.40), 36.75 (±8.66), and 31.8 (±7.46) years, respectively.

The mean time to establish a definitive diagnosis was 5 (±4.24), 6.45 (±4.65), and 5.4 (±3.80) years for groups B, C, and D, respectively. Meanwhile, the mean duration of the disease was 8.5 (±8.43), 10.95 (±7.33), and 9.15 (±6.02) years for the corresponding groups. No statistically significant difference was observed in the average time to establish a definitive diagnosis and the mean duration of disease among groups B, C, and D. Among the 60 patients diagnosed with symptomatic AHP, the predominant subtype was acute intermittent porphyria in 33 (55%) cases, followed by variegate porphyria in 21 (35%) cases. In the group, of asymptomatic AHP, 27 (72.9%) individuals carry a variant in the *HMBS* gene, and 6 (16.2%) have a variant in the *CPOX* gene. Clinical and epidemiological data are summarized in Table 1.

**Table 1:** Clinical and epidemiological data.

**Group A (N = 20)**	
Current age	33.55 (±10.05) years
Gender (M/F)	8 (40%) M/12 (60%) F
**Group B (N = 20)**	
Current age	31.35 (±9.40) years
Age at onset	22.85 (±3.18) years
Time to definitive diagnosis	5.0 (±4.24) years
Duration of disease	8.5 (±8.43) years
Gender (M/F)	4 (20%) M/16 (80%) F
*Subtype of AHP*	
Acute intermittent porphyria	10 (50%)
Variegate porphyria	8 (40%)
Hereditary coproporphyria	2 (10%)
*Clinical manifestation*	
Abdominal pain	5 (25%)
Acute encephalopathy	3 (15%)
Acute flaccid paralysis	10 (50%)
Status epilepticus	2 (10%)
**Group C (N = 20)**	
Current age	36.75 (±8.66) years
Age at onset	25.8 (±5.24) years
Time to definitive diagnosis	6.45 (±4.65) years
Duration of disease	10.95 (±7.33) years
Gender (M/F)	5 (25%) M/15 (75%) F
*Subtype of AHP*	
Acute intermittent porphyria	10 (50%)
Variegate porphyria	7 (35%)
Hereditary coproporphyria	3 (15%)
*Chronic manifestation*	
Gastrointestinal symptoms	10 (50%)
Pain	14 (70%)
Mood/sleep disturbance	14 (70%)
Other symptoms	12 (60%)
**Group D (N= 20)**	
Current age	31.8 (±7.46) years
Age at onset	22.95 (±3.59) years
Time to definitive diagnosis	5.4 (±3.80) years
Duration of disease	9.15 (±6.02) years
Gender (M/F)	3 (15%) M/17 (85%) F
*Subtype of AHP*	
Acute intermittent porphyria	13 (65%)
Variegate porphyria	6 (30%)
Hereditary coproporphyria	1 (5%)
*Chronic manifestation*	
Gastrointestinal Symptoms	14 (70%)
Pain	17 (85%)
Mood/sleep disturbance	17 (85%)
Other symptoms	16 (80%)
**Group E (N = 18)**	
Current Age	36.38 (±10.12) years
Gender (M/F)	6 (33.3%) M/12 (66.6%) F
*Subtype of AHP*	
Acute intermittent porphyria	12 (66.6%)
Variegate porphyria	3 (16.6%)
Hereditary coproporphyria	3 (16.6%)
**Group F (N = 19)**	
Current age	32.15 (±9.17) years
Gender (M/F)	10 (52.6%) M/9 (47.3%) F
*Subtype of AHP*	
Acute intermittent porphyria	15 (78.9%)
Variegate porphyria	1 (5.2%)
Hereditary coproporphyria	3 (15.7%)
**Group G (N = 20)**	
Current age	36.65 (±7.30) years
Age at onset	28.7 (±4.42) years
Duration of disease	7.95 (±6.43) years
Gender (M/F)	12 (60%) M/8 (40%) F

AHP, acute hepatic porphyria; F, female; M, male.

**Figure 1:**
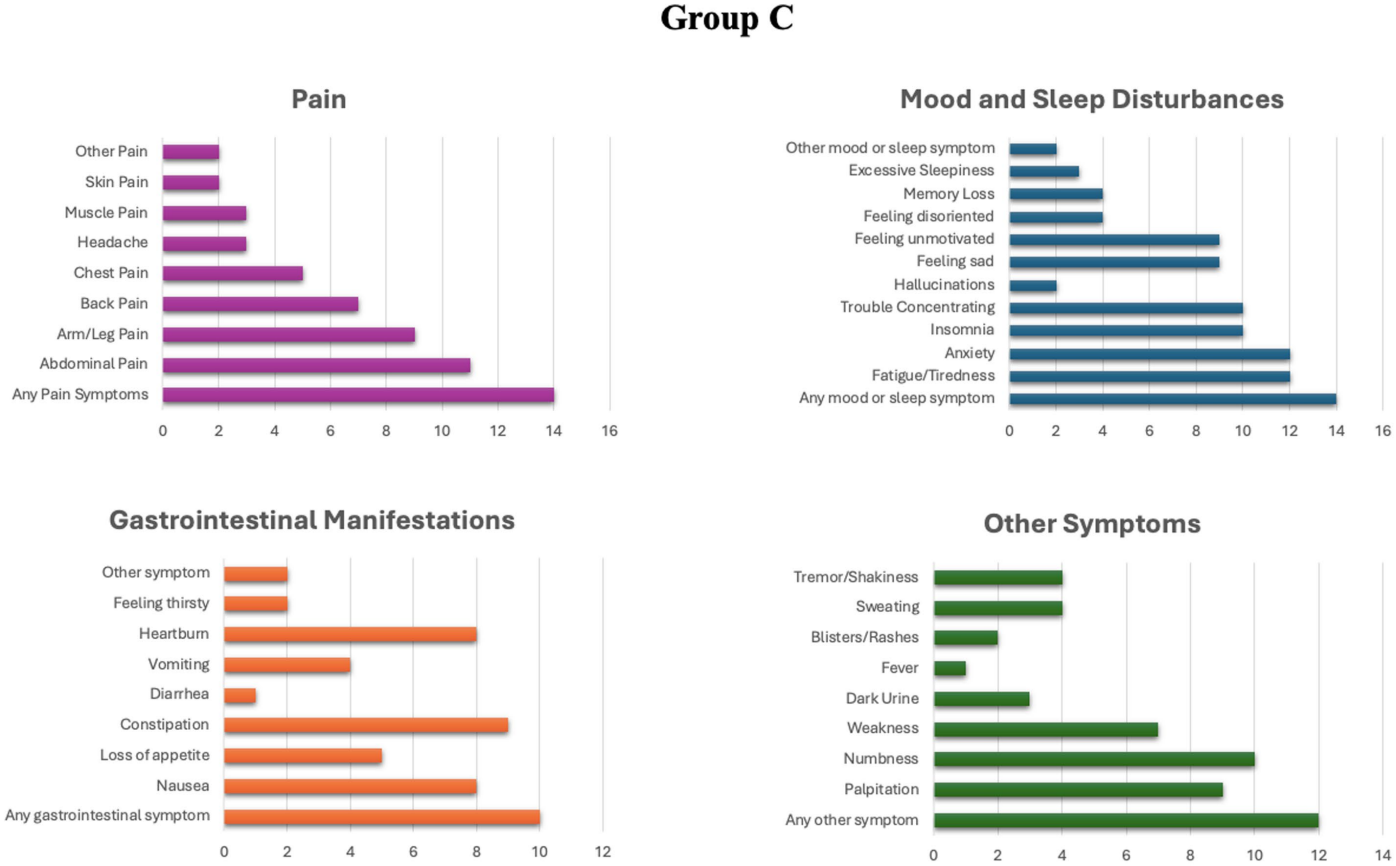
Prevalence of different chronic symptoms in group C.

**Figure 2:**
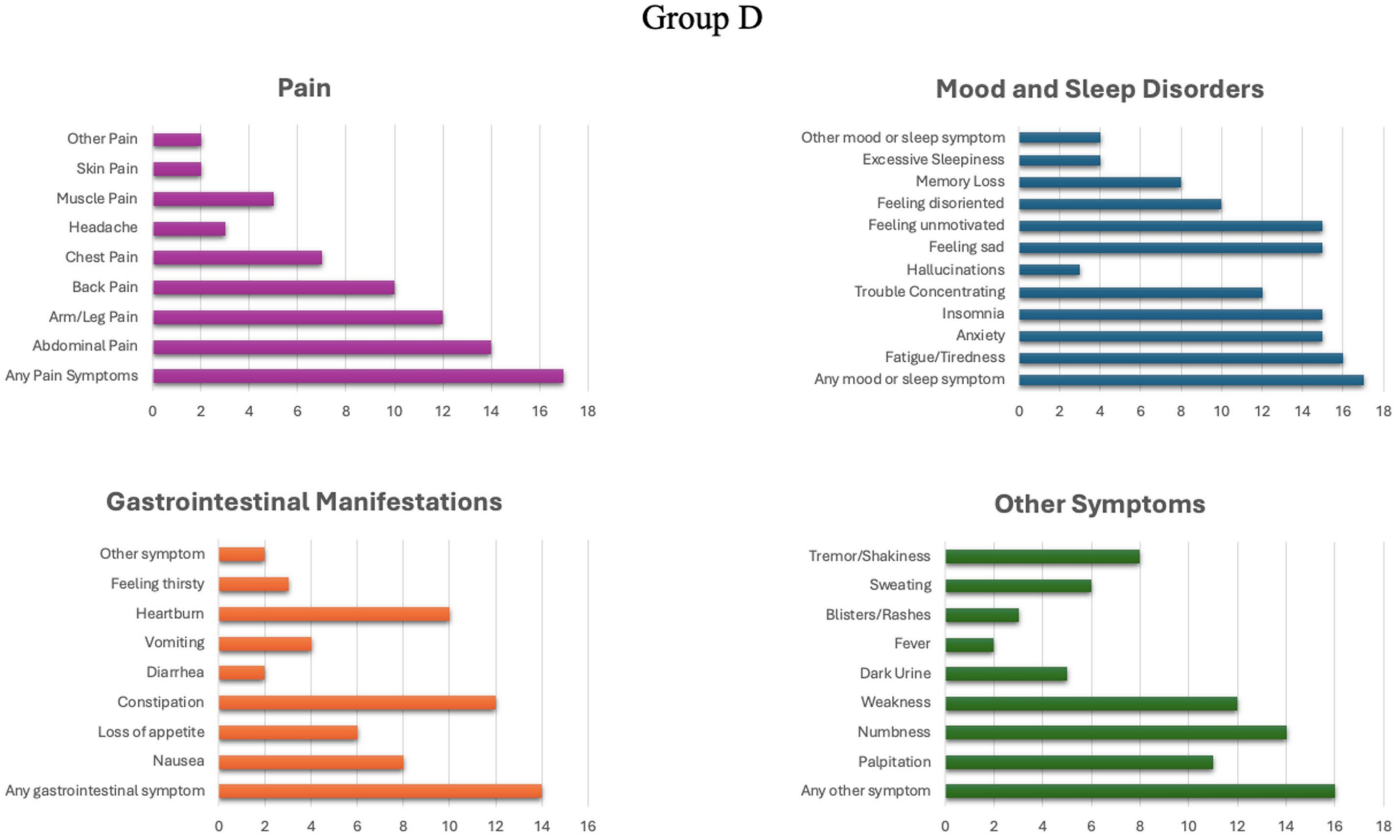
Prevalence of different chronic symptoms in group D.

In group C, 14 individuals (70%) reported the presence of chronic symptoms, while in group D, 17 patients (85%) reported suffering some form of chronic manifestation. The detailed profile of chronic manifestations exhibited by patients in groups C and D is illustrated in Figures 1, 2.

Concerning biochemical tests, patients in group B exhibited a mean of 14.60 (±6.46) mg/g creatinine in ALA levels and 35.78 (±8.48) mg/24 h in PBG levels during the crisis, with 10 (50%) of them manifesting acute flaccid paralysis and 5 (25%) experiencing abdominal pain. The mean ALA levels were 4.03 (±1.03) and 3.63 (±1.49) mg/g of creatinine, while the mean PBG levels were 2.43 (±2.4) and 3.64 (±2.05) mg/24 h for groups C and D, respectively. There is no statistically significant difference in the mean ALA and PBG levels between groups C and D. However, the proportion of individuals with elevated PBG levels in group D is higher, with 14 (70%) individuals, compared to group C, where 5 (25%) out of 20 individuals exhibited elevated PBG levels. Within group E, designated as “high excretors,” the mean levels for ALA and PBG were 6.08 (±0.96) mg/g of creatinine and 4.68 (±1.49) mg/24 h, respectively. All biochemical and clinical assessment data are summarized in Table 2.

**Table 2:** Biochemical and clinical assessment data.

**Group A**	
ALA (mg/g of creatinine)	2.21 (±1.06)
PBG (mg/24 h)	0.97 (±0.30)
NfL (pg/mL)	11.12 (±4.57)
PCS SF-12	51.56 (±4.10)
MCS SF-12	48.50 (±3.31)
**Group B**	
ALA (mg/g of creatinine)	14.60 (±6.46)
PBG (mg/24 h)	35.78 (±8.48)
NfL (pg/mL)	757.09 (±411.91)
**Group C**	
ALA (mg/g of creatinine)	4.03 (±1.03)
PBG (mg/24 h)	2.43 (±2.4)
NfL (pg/mL)	46.09 (±7.92)
PCS SF-12	31.56 (±6.65)
MCS SF-12	30.98 (±6.91)
Number of attacks (life)	3.0 (±1.8)
Number of attacks (last year)	0.50 (±0.68)
PHQ-8	15.45 (±3.35)
GAD-7	17.45 (±3.45)
**Group D**	
ALA (mg/g of creatinine)	3.63 (±1.49)
PBG (mg/24 h)	3.64 (±2.05)
NfL (pg/mL)	78.91 (±18.49)
PCS SF-12	28.91 (±4.80)
MCS SF-12	26.77 (±4.46)
Number of attacks (life)	25.7 (±14.7)
Number of attacks (last year)	6.1 (±1.9)
PHQ-8	18.65 (±3.64)
GAD-7	15.8 (±3.0)
**Group E**	
ALA (mg/g of creatinine)	6.08 (±0.96)
PBG (mg/24 h)	4.68 (±1.49)
NfL (pg/mL)	10.61 (±3.31)
**Group F**	
ALA (mg/g of creatinine)	2.49 (±0.93)
PBG (mg/24 h)	1.21 (±0.29)
NfL (pg/mL)	10.55 (±3.32)
**Group G**	
NfL (pg/mL)	112.28 (±25.06)
PCS SF-12	29.18 (±7.26)
MCS SF-12	32.82 (±8.67)

ALA, delta-aminolevulinic acid; GAD-7, 7-item Generalized Anxiety Disorder scale; MCS, mental component summary; NfL, neurofilament light chain; PBG, porphobilinogen; PCS, physical component summary; SF-12, Social functioning-12 Health Survey; PHQ-8, 8-item Patient Health Questionnaire depression scale.

The mean value (±SD) of neurofilament light chain (NfL) levels for group A to G were 11.12 (±4.57), 757.09 (±411.91), 46.09 (±7.92), 78.91 (±18.49), 10.61 (±3.31), 10.55 (±3.32) and 112.28 (±25.06) pg/mL, respectively. The level of NfL during an attack was on average 68× higher compared to levels observed in normal controls and showed a strong correlation with mean ALA (*R* = 0.83; *p* = 0.005) and PBG levels (*R* = 0.79; *p* = 0.0002) and no correlation with age at disease onset, time to definitive diagnosis and disease duration.

Patients from group were stratified based on the following criteria: (1) clinical manifestations involving the peripheral nervous system (abdominal pain, acute flaccid paralysis) versus manifestations of the central nervous system (status epilepticus, encephalopathy, acute psychosis); (2) age at the time of the attack; (3) duration of the attack in days until neurofilament collection; (4) number of previous attacks. There was no statistically significant difference in neurofilament levels according to the aforementioned stratification criteria. The neurofilament levels were not assessed following therapeutic intervention for the treatment of acute episodes in this study.

**Figure 3:**
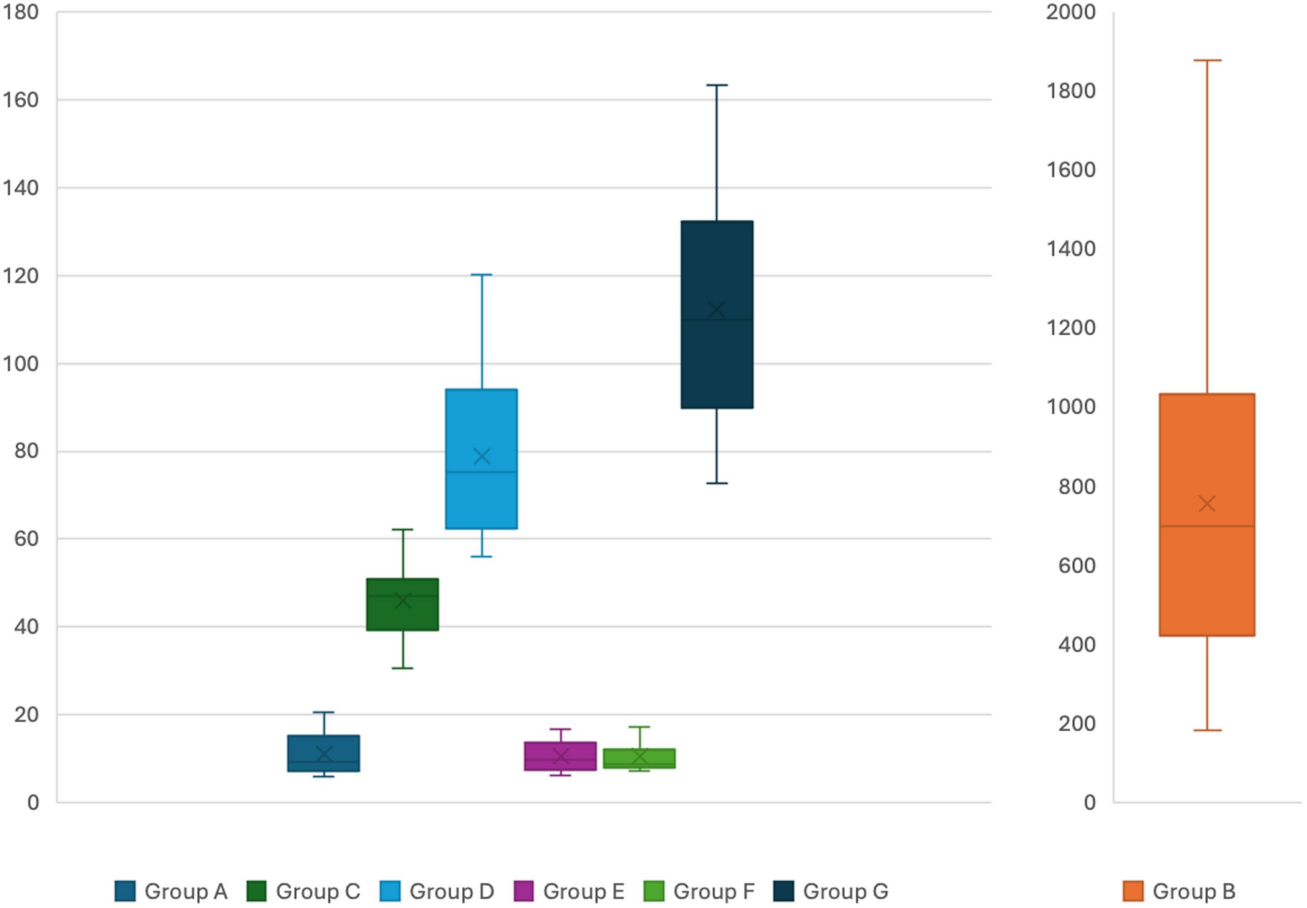
Serum neurofilament light chain levels for the different groups. *Y* axis presents serum levels in pg/mL. Different bar scales are used for group B compared to the other groups.

The patients in groups C and D were stratified according to the following criteria: (1) abnormal findings in neurophysiological studies by nerve conduction studies and electromyography; (2) nature of chronic symptoms (neuropsychiatric, gastrointestinal, and pain); (3) use of opioid medications and pain intensity; (4) current age and duration of the disease. No statistically significant difference in neurofilament levels was observed with the proposed stratification. Levels of NfL for each group are showed in Figure 3.

There is a statistically significant difference in mean NfL levels between individuals in group C (classified as “sporadic attacks”) and healthy individuals in group A, with a mean difference of 34.97 pg/mL (95% CI = 30.83–39.11, *p* = 0.00001). The levels of NfL in group C exhibited a strong correlation with ALA (*R* = 0.92; *p* = 0.005) and PBG levels (*R* = 0.79; *p* = 0.00003). Patients in group D, identified as “recurrent attacks,” demonstrated a mean difference of 67.79 pg/mL (95% CI = 59.16–76.42, *p* = 0.00001) in NfL levels compared to healthy individuals in group A. Additionally, a strong correlation was observed between NfL levels and the number of attacks in the last year (*R* = 0.86; *p* = 0.005), as well as with ALA (*R* = 0.91; *p* = 0.005) and PBG levels (*R* = 0.96; *p* = 0.05).

Among patients in groups C and D, a mean difference of 32.82 pg/mL (95% CI = 23.70–41.93, *p* = 0.00001) was observed in neurofilament levels. Individuals in groups E and F demonstrate mean neurofilament levels comparable to those in normal controls from group A, with no statistically significant difference in neurofilament levels among these three groups.

The mean difference in neurofilament levels between patients with ATTRv-PN and normal controls in group A was 101.1 (95% CI = 89.63–112.69, *p* = 0.00001), and compared to patients in groups B and C, it was 66.19 (95% CI = 54.29–78.08) and 33.37 pg/mL (95% CI = 19.26–47.47, *p* = 0.00001), respectively.

The mean PCS and MCS scores for patients with “sporadic attacks” (group C) were 31.56 (±6.65) and 30.98 (±6.91), respectively, in contrast to scores of 28.91 (±4.80) and 26.77 (±4.46) observed in patients with “recurrent attacks” (group D). The mean difference in the PCS score between patients in group C and healthy controls in group A is −20.00 (95% CI = −23.54 to −16.46, *p* = 0.00001). Similarly, there is a mean difference of −22.92 (95% CI = −25.79 to −20.05, *p* = 0.00001) in the PCS score between patients in group D and those in group A. There is no statistically significant difference in the mean scores for the PCS between patients in groups C and D. However, there is a significant difference of 4.20 (95% CI = 0.47–7.93, *p* = 0.01) in the MCS scores between patients of groups C and D.

For group G, there is a mean difference of −22.37 (95% CI = −26.15 to −18.59, *p* = 0.00001) in the PCS and −15.72 (95% CI = −19.92 to −11.52, *p* = 0.00001) in the MCS compared to group A. However, there is no statistically significant difference in mean PCS scores between groups C, D, and G. In terms of the MCS component, there is a mean difference of −5.99 (95% CI = −10.40 to −1.58, *p* = 0.004) between patients in groups D and G, respectively.

In group C, both the PCS and MCS components of the SF-12 scale exhibited no statistically significant correlation with the age at onset of symptoms, duration of disease, number of attacks during life and in the last year, or with the levels of neurofilament, ALA, and PBG.

A moderate inverse correlation was observed between the PCS component of the SF-12 scale and neurofilament levels (*R* = −0.60; *p* = 0.006), along with the number of attacks in the last year (*R* = −0.54; *p* = 0.01), as well as the levels of ALA (*R* = −0.63; *p* = 0.003) and PBG (*R* = −0.55; *p* = 0.01) for patients with recurrent attacks in group D. Similarly, the MCS component of the SF-12 scale demonstrated a moderate inverse correlation with neurofilament levels (*R* = −0.60; *p* = 0.004), the number of attacks in the last year (*R* = −0.73; *p* = 0.0002), and the levels of ALA (*R* = −0.49; *p* = 0.02) and PBG (*R* = −0.62; *p* = 0.003) in group D.

About neuropsychiatric profile, 17 (85%) patients exhibited moderate to severe depression in group C versus 20 (100%) of those in group D. In group C, the mean score on the PHQ-8 scale was 15.45 (IQR = 13.75–18.25) with a strong inverse correlation of PHQ-8 score and MCS component of SF12 (*R* = −0.96; *p* = 0.005) and a positive strong correlation with GAD-7 (*R* = 0.80; *p* = 0.00002). In group D, the mean score on the PHQ-8 scale was 18.65 (IQR = 16.75–21.25), indicating a positive correlation between the PHQ-8 score and neurofilament levels (*R* = 0.95; *p* = 0.005), the number of attacks in the last year (*R* = 0.79; *p* = 0.00003), ALA (*R* = 0.93; *p* = 0.005) and PBG levels (*R* = 0.90; *p* = 0.005), and with GAD-7 score (*R* = 0.46; *p* = 0.04). Additionally, a moderate inverse correlation of PHQ-8 was observed with the PCS (*R* = −0.64; *p* = 0.003) and MCS (*R* = −0.56; *p* = 0.009) component scores of the SF-12 scale.

Severe anxiety, indicated by a GAD-7 score higher than 15, was observed in 17 (85%) of patients in group C compared to 13 (65%) in group D. In group C, the PHQ-8 score demonstrated a strong inverse correlation solely with the MCS component of the SF-12 scale (*R* = −0.82; *p* = 0.005). Conversely, for group D, there was a moderate correlation between the GAD-7 score and NfL levels (*R* = 0.54; *p* = 0.01), the number of attacks in the last year (*R* = 0.67; *p* = 0.001), and PBG levels (*R* = 0.60; *p* = 0.005). Additionally, an inverse correlation was observed between the GAD-7 score and the PCS (*R* = −0.47; *p* = 0.03) and MCS (*R* = −0.86; *p* = 0.005) components of the SF-12 assessment.

## 4 Discussion

Currently, a significant and unresolved debate exists in the literature regarding the natural progression of patients with Acute Hepatic Porphyria. There is ongoing uncertainty surrounding the origin and mechanisms underlying the chronic symptoms observed in patients, leading to a dichotomy in the classification of patients into “sporadic attacks” and “recurrent attacks.” This classification dilemma has contributed to several uncertainties regarding the optimal medical management and treatment strategies for these individuals (6, 8, 21).

Lately, a growing body of research has highlighted that individuals with AHPs experiencing sporadic attacks face substantial declines in their quality of life and exhibit a high frequency of chronic symptoms, resulting in a notable medical and economic burden (7, 9, 17, 22–24).

Our data support recent findings by indicating that the prevalence of chronic symptoms is comparable among patients experiencing recurrent and sporadic attacks. Additionally, the quality of life for individuals in both groups (C and D) is diminished in comparison to normal controls. Importantly, there is no significant difference in the PCS and MCS quality of life scores in relation to the frequency of attacks. Furthermore, a noteworthy observation is that patients with AHPs, irrespective of the number of attacks, exhibit a reduction in quality of life of a similar magnitude to individuals with other forms of progressive hereditary neuropathy, such as ATTRv.

The results also indicate that NfL levels during an attack can be up to 10 times higher than those observed in patients with AHP outside of the crisis period. This reinforces the role of acute axonal damage as the underlying cause of the manifestations observed during the crises (3, 25, 26), suggesting that NfL analysis may serve as a promising biomarker to indicate the onset of an acute episode of the disease along with the established biochemical analysis of porphobilinogen elevation, already established in the literature as the gold standard. The use of NfL analysis as a biomarker for acute attacks may aid in identifying acute neurological damage in patients with AHPs. Additionally, it may prove useful in distinguishing an attack from an exacerbation of a pre-existing symptom, particularly in the context of patients with chronic symptoms who maintain elevated levels of PBG (considered the gold standard biomarker for characterizing an attack in AHPs) (2).

This study additionally demonstrates that individuals categorized as “high excretors” do not exhibit elevated levels of neurofilament compared to normal controls. This implies that certain individuals can sustain high levels of ALA and PBG without displaying axonal damage, indicating that other molecular and pathological events must transpire for these patients to manifest clinical symptoms.

Our findings also reveal that patients with AHPs, whether classified based on the frequency of attacks as recurrent or sporadic, exhibit elevated levels of neurofilament compared to normal controls, indicating axonal injury and progressive neurological damage. In patients with recurrent attacks, the strong correlation between neurofilament levels and the PCS and MCS components of the SF-12 quality of life scale, as well as with psychiatric assessment instruments such as the PHQ-8 and GAD-7, suggests a higher degree of neurological damage in this group.

For patients with sporadic attacks, neurofilament levels did not exhibit a statistically significant correlation with quality-of-life scores and psychiatric assessment. Despite this, the increased biomarker levels compared to healthy controls indicate underlying progressive neurological damage in this patient subgroup. Therefore, consideration should be given to treatment strategies to prevent the occurrence of acute attacks that may exacerbate neurological impairment.

Recently, with the worldwide approval of givosiran, a therapy based on small interfering RNA (siRNA), there has been a significant advancement in the treatment landscape for AHP. Givosiran, an N-acetyl-D-galactosamine-conjugated siRNA, is designed to specifically target ALAS1 messenger RNA in the liver. The consolidated long-term results of this therapeutic intervention have demonstrated its effectiveness in reducing the frequency of attacks in patients with “recurrent attacks.” Furthermore, it has exhibited the ability to decrease the need for prophylactic hemin treatment, resulting in lower hospitalization rates and an overall improvement in the quality of life (27–29). Despite these achievements, there remains limited knowledge and discussion regarding the impact of givosiran on the chronic manifestations of the disease and its benefits for patients with “sporadic attacks.”

The study has some limitations that deserve attention, such as the small sample size per group, making it difficult to make inferences for larger populations. There is also a potential selection bias since patients were recruited from a specialized neurological reference center. Patients monitored at this center typically exhibited severe neurological manifestations throughout the course of the disease, such as acute flaccid paralysis, acute encephalopathy, status epilepticus, and reversible cerebral vasoconstriction syndrome. According to the literature, these manifestations represent approximately 20% of all AHP cases. Therefore, conducting additional studies in large international cohorts with diverse clinical presentation profiles would be beneficial to confirm the role of neurofilament as a biomarker for AHP. The cross-sectional design of the study also prevents establishing longitudinal inferences regarding the behavior of this biomarker in patients during attacks and in the context of chronic manifestations. Additionally, the variation of NfL in patients under therapeutic intervention with heme therapy or givosiran was not evaluated. However, the results presented indicate that NfL shows potential to be explored and evaluated in future studies as a biomarker for AHP in patients undergoing longitudinal follow-up and subjected to different therapeutic interventions.

## 5 Conclusion

This study represents the first in the literature to establish NfL as a biomarker for AHPs. It not only underscores that neurological damage associated with the disease can manifest in any patient, irrespective of the number of attacks but also reinforces the progressive and profoundly debilitating nature of symptoms observed in individuals with AHPs. Additionally, the findings affirm recent data indicating a high prevalence of patients experiencing chronic symptoms and significant impairment in quality of life, irrespective of the frequency of attacks over the course of the disease.

Our findings further contribute to the understanding of neurofilament as a sensitive biomarker for detecting axonal damage, albeit with limited specificity (30, 31). Moreover, they suggest that acute hepatic porphyrias exhibit an underlying progressive degenerative mechanism that can be tracked through neurofilament levels, paralleling observations in both common neurological disorders (such as amyotrophic lateral sclerosis, Alzheimer’s disease, multiple sclerosis, Parkinson’s disease) and rare conditions (such as Guillain–Barré syndrome, progressive supranuclear palsy, CADASIL, adrenoleukodystrophy and hereditary transthyretin-mediated amyloidosis) (32–40).

## Data availability statement

The datasets presented in this article are not readily available because legal restriction apply to this data type. Requests to access the datasets should be directed to pvsgobbi@gmail.com.

## Ethics statement

The studies involving humans were approved by CEP UNIFESP-HSP (CAAE: 50517321.2.0000.5505). The studies were conducted in accordance with the local legislation and institutional requirements. The participants provided their written informed consent to participate in this study.

## Author contributions

PS: Conceptualization, Data curation, Formal analysis, Funding acquisition, Investigation, Methodology, Project administration, Resources, Supervision, Validation, Visualization, Writing – original draft, Writing – review & editing. PdLS: Conceptualization, Formal analysis, Investigation, Methodology, Resources, Validation, Visualization, Writing – original draft, Writing – review & editing. BB: Conceptualization, Formal analysis, Methodology, Resources, Visualization, Writing – original draft, Writing – review & editing. IF: Conceptualization, Methodology, Resources, Visualization, Writing – original draft, Writing – review & editing. HO: Conceptualization, Formal analysis, Methodology, Resources, Validation, Visualization, Writing – original draft, Writing – review & editing. AB: Conceptualization, Investigation, Methodology, Resources, Visualization, Writing – original draft, Writing – review & editing. CP: Conceptualization, Methodology, Resources, Validation, Visualization, Writing – original draft, Writing – review & editing. VM: Conceptualization, Investigation, Methodology, Resources, Validation, Visualization, Writing – original draft, Writing – review & editing. ÍP: Conceptualization, Formal analysis, Investigation, Resources, Validation, Visualization, Writing – original draft, Writing – review & editing. AO: Conceptualization, Formal analysis, Investigation, Methodology, Resources, Validation, Visualization, Writing – original draft, Writing – review & editing. WP: Conceptualization, Formal analysis, Investigation, Methodology, Resources, Supervision, Validation, Visualization, Writing – original draft, Writing – review & editing.
